# Zoonotic tuberculosis knowledge and practices among cattle handlers in selected districts of Bangladesh

**DOI:** 10.1371/journal.pntd.0009394

**Published:** 2021-04-30

**Authors:** Sk Shaheenur Islam, Tanzida Begum Rumi, S. M. Lutful Kabir, A. K. M. Anisur Rahman, Md. Mahmudul Hasan Faisal, Robiul Islam, Adri G. M. van der Zanden, Michael P. Ward, Allen G. Ross, Zeaur Rahim

**Affiliations:** 1 Department of Livestock Services, Krishi Khamar Sarak, Dhaka, Bangladesh; 2 Department of Microbiology and Hygiene, Bangladesh Agricultural University, Mymensingh, Bangladesh; 3 International Centre for Diarrhoeal Disease and Research, Bangladesh (icddr,b), Dhaka, Bangladesh; 4 Department of Medicine, Bangladesh Agricultural University, Mymensingh, Bangladesh; 5 Department of Microbiology, Jagannath University, Dhaka, Bangladesh; 6 LAB MICTA, Hengelo, The Netherlands; 7 Sydney School of Veterinary Science, The University of Sydney, Camden, New South Wales, Australia; University of Sharjah, UNITED ARAB EMIRATES

## Abstract

We assessed zoonotic tuberculosis (zTB) knowledge and prevention and control practices of 404 cattle handlers via a survey in three dairy-intensive districts of Bangladesh. Most respondents were aged 30–49 (52%) and male (95%). Almost all (99%) recognized the important public health burden of tuberculosis in Bangladesh, however, most (58%) had inadequate knowledge about zTB transmission to humans. Inappropriate practices such as: not using protective equipment (98%); smoking, drinking or eating food whilst working with cattle (69%); and sharing the same premises with animals (83%) were identified. Cattle handlers educated at secondary or higher levels were 2.82- (95% CI: 1.59–5.10) and 5.15 times (95% CI: 1.74–15.20) more likely to have adequate knowledge of control and prevention activities compared to those with no formal education. Those who had reared animals for 1–5 years were 2.67 times (95% CI: 1.44–4.91) more likely to have adequate knowledge, compared to those who reared animals for >15 years. Cattle handlers with a monthly incomes of 10,000–20,000 taka were significantly (Odds Ratio = 0.36, 95% CI: 0.14–0.92) less likely to have adequate knowledge compared to those with monthly incomes <10,000 taka. Cattle handlers with high school or higher education were 6.98 times (95% CI: 2.47–19.71) more likely to use appropriate zTB control and prevention practices compared to those without formal education. Those who had reared animals for 1–5 years, 6–10 years and 11–15 years were 2.72- (95% CI: 1.42–5.24), 2.49- (95% CI: 1.29–4.77) and 2.86 times (95% CI: 1.13–7.23) more likely to apply appropriate practices compared to those who reared animals for >15 years. Overall, education, duration of cattle rearing and monthly income predicted zTB knowledge and practices. There is an urgent need to educate those at high-risk of zTB transmission on issues including the handling of infected animals, and general hygiene. A One Health approach, to support the Sustainable Development Goals and the End TB strategy, appears to be the way forward.

## Author summary

Zoonotic tuberculosis (zTB) is a form of tuberculosis derived mainly from cattle (bovine tuberculosis, bTB) via direct or indirect contact (inhalation), and/or consumption of contaminated animal products or water (ingestion). We evaluated the zTB knowledge and practices among cattle handlers in selected districts of Bangladesh via a questionnaire. It was observed that handlers are moderately aware of tuberculosis as an important public health burden in Bangladesh. However, they have inadequate knowledge on the transmission of zTB from humans and animals. Most of the cattle handlers do not use personal protective equipment. Cattle handlers often smoke, drink or eat whilst working with cattle and some even share the same premise with animals. Education, duration of cattle rearing and cattle handlers’ monthly income significantly influenced cattle handlers’ zTB knowledge and practices. Based on results of this study we recommend implementation of a One Health platform in order to enable health education programs targeting at risk farmers.

## Introduction

Tuberculosis (TB) is one of the world’s most important infectious diseases, with 10 million new cases and 1.2 million deaths in 2019 alone [1]. It is a major public health problem in Bangladesh [2]. Bangladesh is one of 30 high TB burden countries, with an estimated annual incidence rate of 361 per 100,000 in 2019, which represents 3.6% of the total global incidence [1].

Zoonotic TB (zTB) is a form of tuberculosis in humans predominately caused by *Mycobacterium bovis*, but to a lesser extent by *M*. *tuberculosis*, *M*. *caprae* and *M*. *orygis* (*Mycobacterium tuberculosis* complex, MTC) [3, 4]. *M*. *bovis* causes chronic TB in cattle (bovine TB, bTB), however, it may cause infection in goats and other mammalian species [5], impacting milk and meat production in these animals [6, 7]. Humans can be infected with zTB via direct contact with infected animals, airborne transmission or by consuming contaminated raw milk or meat [8]. Specific groups such as veterinarians, farmers, cattle handlers, slaughterhouse workers, and butchers are at occupational risk for zTB [9, 10].

Globally, an estimated 140,000 new cases and 11,400 deaths occurred due to zTB in humans in 2019. In the Southeast Asia region (which includes Bangladesh) there were 43,400 cases and 2,020 deaths [1]. However, the full burden of zTB is unknown and is likely to be grossly underestimated, particularly in low and middle income countries (LMICs) where limited epidemiologic information is available [11–13]. In Bangladesh, the impact of zTB on human health has also been underestimated in the national tuberculosis programme [14]. The overall animal level prevalence of bTB has been estimated to range from 2–11.3% [15–19]. Bangladesh submits regularly (six monthly or yearly) disease information on the presence of zTB (MTC) in cattle in accordance with the World Organization for Animal Health criteria [20]. These yearly reports revealed that the disease is endemic in animals. zTB has a dual impact on human health and livestock production. Human populations having direct or close contact with livestock enables transmission. Indirect impacts on animal health include reduced milk and meat production intensifying poverty in marginalized communities [21, 22].

Bangladesh is one of the most densely populated countries in the world (1240 people / sq. km of land area) [23] with the most dense ruminant population (145 large ruminants/ sq. km in 2010 [24]. People live in a close interface with animals and some farmers even share the same premises with livestock. The consumption of raw milk is rare but not uncommon. At present milk processors in Bangladesh use Ultra Heat Treatment (UHT) to pasteurize 1 million liters of fresh milk daily for the local consumers [25]. However, the current milk pasteurization system is not adequate to meet the total production demand. Therefore, the transmission of milk-borne pathogens, especially extra-pulmonary TB (EPTB) through the consumption of milk is enormous [26]. In addition, slaughtering activities lack safety and personal hygiene compliance measures that block zoonotic transmission of bTB to humans. To meet the increasing demand for milk the number of high yielding crossbred cattle is also increasing. These exotic breeds are more susceptible to bTB thus potentiating zTB transmission [27].

In Bangladesh limited research has been conducted to estimate the burden of bTB in source animals and to determine its zoonotic implications. Control and prevention of infectious diseases depend greatly on people’s knowledge and perceptions [28, 29]. Occupationally exposed individuals (cattle handers or farmers) have been grossly neglected and their bTB knowledge and practices have not been evaluated. Given this fact we explored the level of zTB knowledge and practices and their associated risk factors among cattle handlers and farmers for the first time in selected cattle intensive districts of Bangladesh. Information relating to zTB knowledge and practices in high risk groups remains a critical prerequisite to design an evidence-based control programme to enhance TB surveillance and ultimately fulfilling the End-TB strategy in Bangladesh by 2030.

## Materials and methods

### Ethical statement

The study was approved by the ethics review board (ERD) of International Center of Diarrhoeal Disease and Research (icddr,b) as a part of a larger project (ID No # PR 17121). Additionally, ethics approval was received from the Animal Welfare and Experimentation Ethics Committee (AWEEC) of Bangladesh Agricultural University (AWEEC/BAU/2019(24)). All respondents included in this study were adult (≥ 18 years). All participants were briefed on the objectives and benefit of the study. Oral consent was obtained as a substantial number of participants could not read and write. The information provided by the respondents was made confidential by using unique code numbers instead of names. Privacy and the opportunity to withdraw from the study at any time without prejudice were guaranteed and aligned with the Declaration of Helsinki [30].

### Methods

#### Study area, design and population

**Fig 1:**
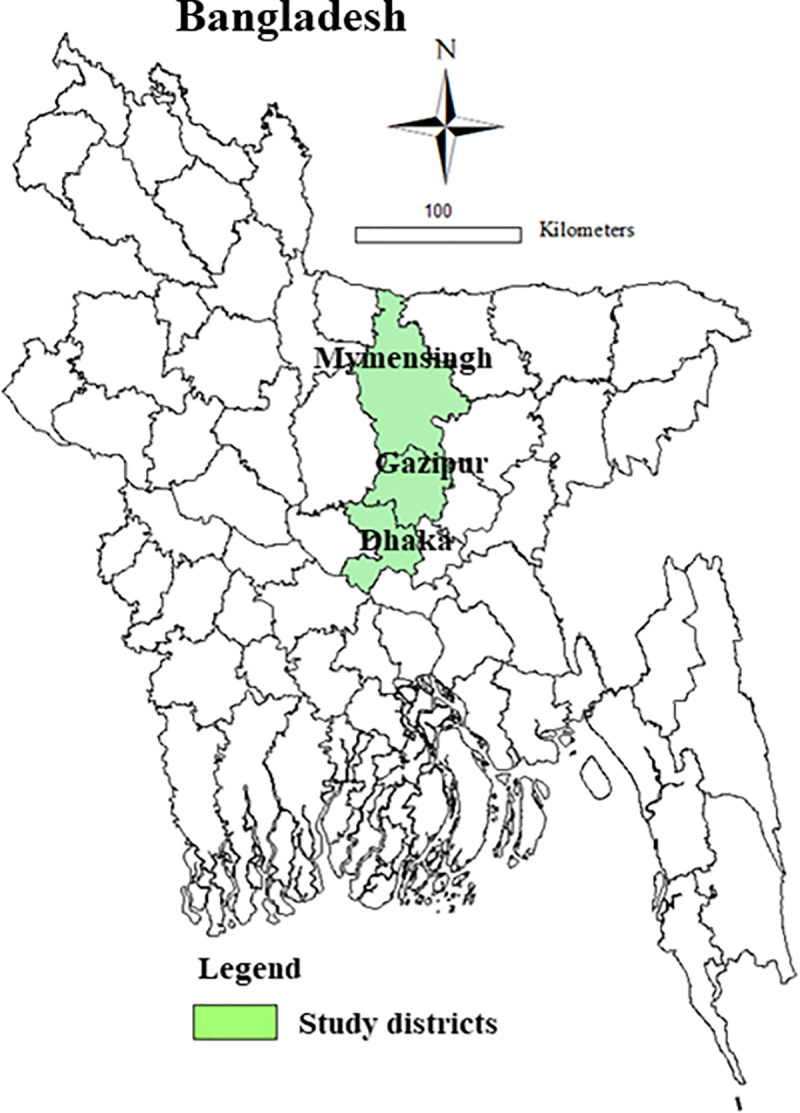
Map of the study districts (Dhaka, Gazipur and Mymensingh) in Bangladesh. The map was generated in ArcGIS-ArcMap version 10.3 (ESRI Co., Redlands, California, USA) using geographic coordinates of the study districts captured through Garmin eTrex 10.

The study was conducted in Dhaka and Mymensingh districts, Bangladesh (Fig 1). Dhaka and Gazipur districts are located in the Dhaka division in the central zone of the country. Dhaka is the most densely populated district in the nation. Due to the large demand for fresh milk in the capital, these three districts are considered to be promising cattle rearing sites. A total of 344 321 heads of crossbred farmed cattle are present in these three districts [31]; of these nearly 52% are milking cows [19].

A cross-sectional survey was conducted to assess the knowledge and practices of cattle handlers on zTB from November 2018 to December 2019. A list of dairy farms from the three relevant district livestock offices (Dhaka, Gazipur and Mymensingh) was obtained. The list of the farms was entered into a Microsoft spreadsheet (Microsoft Excel 2010) and used as the sampling frame. A random number was generated for each farm using the “rand” function of Microsoft Excel. Initially farms were selected proportionately from the three districts. Then one cattle handler from each selected farm who met the inclusion criteria was selected. The participants were included as per the inclusion criteria: a cattle handler must be the owner of the farm (farmer) or an animal attendant with an age ≥ 18 years. A cattle handler was defined as an individual who is involved in rearing/feeding/drinking/caretaking, or having direct or indirect contact with crossbred farmed cattle at least daily.

#### Sample size calculation and sampling method

The sample size for the cattle handlers was estimated using the following formula [32]:

$$n=Z^{2}p(1-p)/d^{2}$$

Where n denotes the required sample size; Z^2^, the Z-score at 95% confidence interval (1.96); p, the expected frequency of likely knowledge and practices on bTB among cattle handlers (0.50); and d, the expected margin of error (0.05). The formula with the above assumptions yielded a sample size of 385. However, a contingency of 5% was included to account for non-responsive participants, and the final sample size was 404 participants from the three districts.

#### Questionnaire design and data collection

A pretested semi-structured questionnaire was designed (S1 Text) containing 32 questions, mostly close-ended, to facilitate data management, minimize inconsistency and increase precision of responses [32]. The questionnaire had 3 sections. The first section included information on the cattle handlers’ demographic and socio-economic characteristics (9 questions) comprising: location, age, gender, religion, occupation, income, family size, education status, and duration of cattle rearing. The second section contained 13 questions to assess the handlers’ knowledge and the third section, with 10 questions, was used to assess the prevention and control measures practiced by the cattle handlers.

The questionnaire was developed in English and translated into Bengali language. Paraphrasing of the questions with local dialect was made during questionnaire administration for the respondents who do not have a formal education. A team consisting of an expert veterinarian, a physician and two trained enumerators were involved in questionnaire administration and to collect data from the participants during the pretesting and the main survey. Of the three sections of the questionnaire, data on sociodemographic status (first section) were captured through field interviews and the content used for further analysis. However, only correct responses were considered for determining level of knowledge (1—adequate and 0—inadequate) and appropriateness of practices (1—appropriate and 0—inappropriate) and a minimum of sixty percent (60%) correct answers were regarded as respondents’ adequate knowledge or appropriate practice (S1 and S2 Data).

#### Data management and statistical analysis

Data on knowledge and practices were recorded as hard copies and then entered into a Microsoft Excel 2010 (MS excel) (Microsoft Corp, Redmond WA, USA) spread sheet. The data set was coded, checked for integrity and exported to R 3.6.0 [33] for analysis.

*Pearson chi-square test*. The cattle handlers’ demographic and socio-economic factors associated with zTB knowledge and practices were analysed separately. Continuous variables such as age, duration of animal rearing and monthly income were converted into categorical variables for analysis. The cattle handlers’ knowledge (adequate versus inadequate) and practices (appropriate versus inappropriate) on zoonotic tuberculosis as the outcome and their demographic and socio-economic characteristics were used as explanatory variables. Pearson chi-square test was used to assess the association between cattle handlers’ knowledge and practices and their demographic and socio-economic characteristics. The R functions “table” and “chisq.test” were used to construct contingency tables and to perform chi-square tests, respectively. Any explanatory variable associated with knowledge and practices with a p-value of ≤ 0.20 was selected for multiple logistic regression analysis. Collinearity among explanatory variables was assessed by Cramer’s phi-prime statistic (R package “vcd,” “assocstats” functions). A pair of variables was considered collinear if Cramer’s phi-prime statistic was >0.70 [34].

*Multivariable logistic regression analyses*. Separate stepwise multiple logistic regression models were used to identify demographic and socio-economic factors associated with zTB knowledge and practices. The final multivariable models were automatically selected based on the lowest Akaike’s information criterion (AIC) value. Hosmer-Lemeshow goodness-of-fit tests [35] using the “hoslem.test” function of the R package “ResouceSelection” [36] was used to assess the overall model fit. The validity of the final models was checked by the k-fold cross-validation method by the “confusionMatrix” function of the R package “caret” [37]. The models’ ability to discriminate cattle handlers’ adequate versus inadequate knowledge and appropriate versus inappropriate practices were further evaluated by constructing receiver operative characteristic (ROC) curves using the “roc” function of the R package “pROC”[38]. Model diagnostics were performed by plotting residuals against predictors or fitted values using “residualPlots” function [39]. Outliers were examined by “outlierTest” function [39]. Confounding was checked by observing the change in the estimated coefficients of the variables that remained in the final model by adding a non-selected variable to the model. If the inclusion of this non-significant variable led to a change of more than 25% of any parameter estimate, that variable was considered to be a confounder and retained in the model [40]. The two-way interactions of all variables remaining in the final model were assessed for significance based on AIC values, rather than significance of individual interaction coefficients [40].

## Results

### Demographic and socio-economic characteristics of the respondents

Most of the participants (78.7%, n = 318) included were from Dhaka district. More than 50% (n = 206) were cattle farmers. Only 7.2% (n = 109) participants were Hindu and 94.8% (n = 383) were male. Approximately one third (33.7%, n = 136) had been rearing cattle for 1–5 years and 32% had a monthly family income of BDT 10,000–20,000. Nearly a third of participants (n = 122, 30.2%) had no formal education (Table 1).

**Table 1:** Demographic and socio-economic characteristics of cattle handlers (N = 404) in the study districts of Bangladesh.

Characteristics	Category	Frequency (n)	Percentage (%)
Location (Districts)	Dhaka	318	78.7
	Gazipur	29	7.2
	Mymensingh	57	14.1
Age (years)	20–29	62	15.3
	30–39	109	27.0
	40–49	102	25.2
	50–59	88	21.8
	≥ 60	43	10.6
Sex	Male	383	94.8
	Female	21	5.2
Religion	Muslim	375	92.8
	Hindu	29	7.2
Occupation	Cattle farmers	206	51.0
	Animal attendants	64	15.8
	Other profession (business man/shop keeper/service holder)	134	33.2
Monthly income in BDT (N = 334)*	<10000	28	6.9
	10,000–20,000	129	31.9
	>20,000–50,000	120	29.7
	>50000	57	14.1
Education	No Formal education	122	30.2
	Basic education(primary)	108	26.7
	Secondary(six to ten)	121	30.0
	Higher secondary	31	7.7
	Graduation and above	22	5.4
Family size	1–2	16	4.0
	3–5	184	45.5
	>5	204	50.5
Duration of animal husbandry (years)	1–5	136	33.7
	6–10	135	33.4
	11–15	33	8.2
	> 15	100	24.8

* 70 respondents did not provide information on monthly income and 1USD = 85 BDT (approximately)

### Knowledge and practices of zoonotic tuberculosis prevention and control

**Fig 2:**
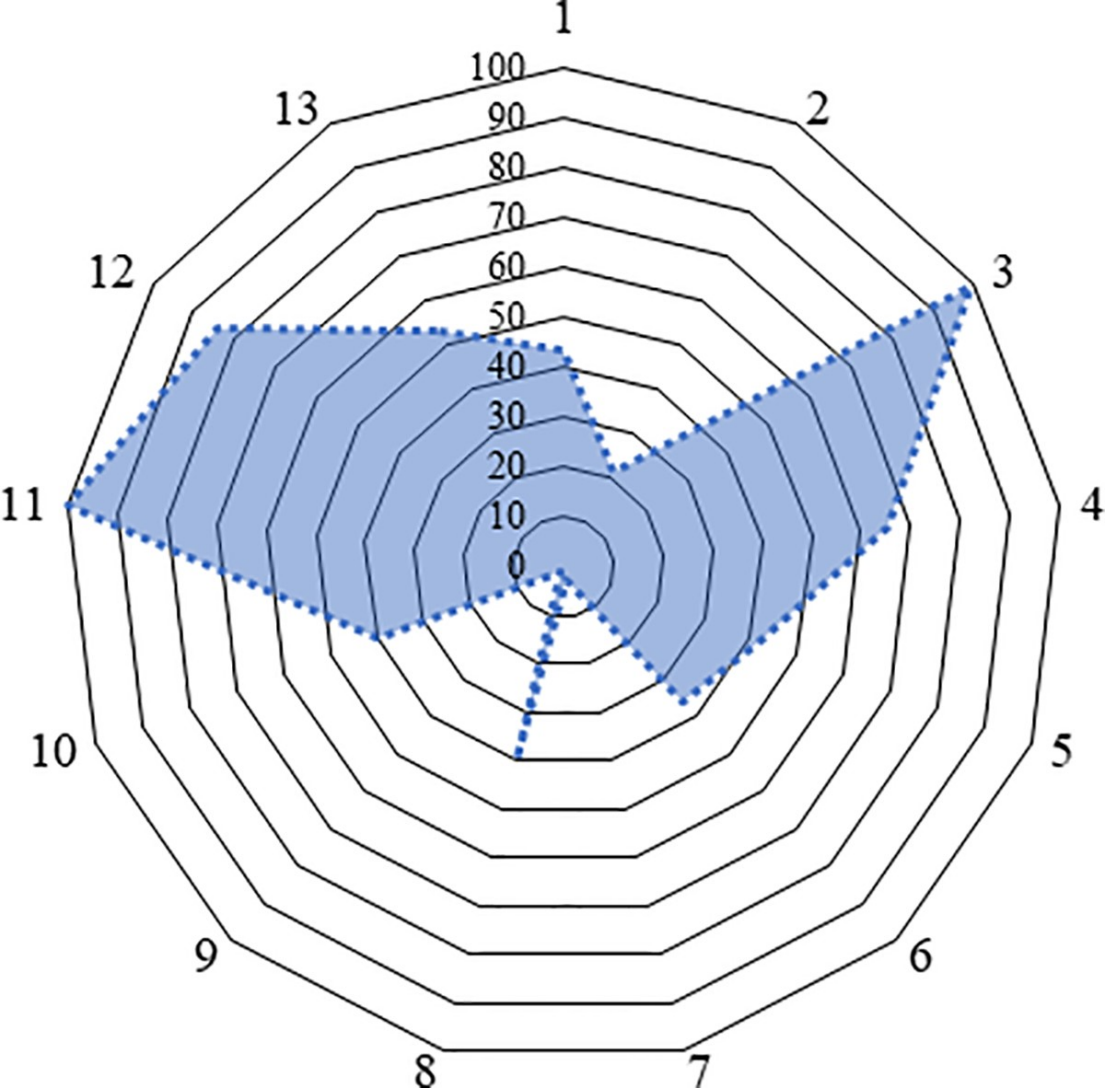
Knowledge assessment of cattle handlers for different questions in the study questionnaire. [(1) Do you know tuberculosis (TB) can be transmitted from animals to humans or humans to animals as a zoonosis? (2) What is the causal agent of TB? (Virus/bacteria/fungus/ other), (3) Do you know tuberculosis is an important public health problem in Bangladesh (Yes/No), (4) What are the symptoms of TB in humans? (Fever/swollen lymph glands /respiratory distress/coughing: ≥2 weeks)/ Others)(at least two), (5) How do humans get infected with TB? (at least two) (consumption of raw/ improper boiled milk/ contact with infected animal (through aerosol)/contact with another infected person/butchering of infected animal/ contact/ consuming raw meat/improper cooked meat of infected animal/ others), (6) Are animals, including wild species, at risk for TB infection? (Yes/No), (7) What are the symptoms of TB in animals? (at least two) (fluctuation of temperature/progressive emaciation/rough hair coat/chronic coughing /presence of dyspnea/ presence of nasal discharge/enlargement of lymph node), (8) Why do you think that your cattle are not at risk of becoming sick by this disease? (maintaining bio-security/ regularly vaccination/ do not let them out of shed/conduct tuberculin skin tests (TST) and to exclude positive reactors from the herd), (9) Do you know animal movement/ purchase of a new animal is responsible for TB transmission in your farm? (Yes/No), (10) Will the boiling of milk kill the milk borne pathogen including the TB bacteria? (Yes/No), (11) Is zoonotic TB in humans curable? (Yes/ No), (12) What type of treatment is available for TB? (modern/traditional /religious believes) and (13) Do you know TB treatment is free of cost and available at the DOTS centers located throughout the country? (Yes/No)].

There were 172 (43%) respondents found to have adequate knowledge on zoonotic tuberculosis prevention and control (S1 Data). The distribution of adequate knowledge among cattle handers for different questions are presented in Fig 2.

Adequate level of knowledge was higher among the other professionals (businessman, shop owner or service holder) (49.3%, n = 66) involved in cattle rearing. However, cattle farmers (39.3%, n = 81) and farm workers (39.1%, n = 25) had nearly similar level of knowledge on zTB prevention and control (Table 2).

**Table 2:** Association between cattle handlers’ demographics, socioeconomic characteristics and overall knowledge of zoonotic tuberculosis in the selected districts of Bangladesh.

Variable	Category	Knowledge		Chi-square
		Inadequate (%)	Adequate (%)	P-value
Sex	Male	222 (58)	161 (42)	0.48
	Female	10 (47.6)	11 (52.4)	
Age	20–29	39 (62.9)	23 (37.1)	0.05
	30–39	64 (58.7)	45 (41.3)	
	40–49	50 (49)	52 (51)	
	50–59	47 (53.4)	41 (46.6)	
	>60	32 (74.4)	11 (25.6)	
Education	No formal	86 (70.5)	36 (29.5)	<0.001
	Basic/Primary	73 (67.6)	35 (32.4)	
	Secondary	52 (43.0)	69 (57.0)	
	Higher secondary	14 (45.2)	17 (54.8)	
	Graduation and above	7 (31.8)	15 (68.2)	
Duration of animal rearing (years)	1–5	62 (45.6)	74 (54.4)	<0.001
	6–10	77 (56.2)	60 (43.8)	
	11–15	24 (77.4)	7 (22.6)	
	> 15	69 (69.0)	18 (31.0)	
Occupation	Others(business man, shop owner, service holder)	68 (50.7)	66 (49.3)	0.16
	Cattle farmers	125 (60.7)	81 (39.3)	
	Farm worker	39 (60.9)	25 (39.1)	
Monthly income (BDT) (N = 334)	<10000	13 (46.4)	15 (53.6)	**<0.001**
	10000–20000	89 (69.0)	40 (31.0)	
	20001–50000	52 (43.3)	68 (56.7)	
	>50000	27 (47.4)	30 (52.6)	

Most of the respondents (98.8%, n = 399) acknowledged tuberculosis as an important public health problem in Bangladesh. More than 65% (n = 265) of respondents correctly confirmed the symptoms of TB in humans, however, most of them (97.3%, n = 393) did not distinguish those in animals. About 40% of respondents (n = 161) confirmed that boiling can kill milk-borne pathogens including TB. All respondents had adequate knowledge that TB is a curable disease and the majority (84.4%, n = 341) preferred treatment of human TB. However, only 53.5% of the respondents knew about the modern treatment of tuberculosis at the directly observed treatment, short-course (DOTS) centers located throughout the country. Most of the respondents (99%, n = 400) had no knowledge of the transmission of zTB through purchase of new animals or animal movement activities (Table 3). Our study confirmed that education, duration of animal rearing and monthly income were associated with adequate knowledge of prevention and control of zTB (Table 2).

**Table 3:** Summary responses for cattle handlers’ knowledge with respect to zoonotic tuberculosis in the selected districts of Bangladesh (N = 404).

Questions/statements	Knowledge	
	Adequate (%)	Inadequate (%)
Do you know tuberculosis (TB) can be transmitted from animals to humans or humans to animals as zoonosis?	174 (43.1)	230(56.9)
What is the causal agent of TB? (virus/bacteria/fungus/ other)	87(21.5)	317(78.5)
Do you know tuberculosis is an important public health problem in Bangladesh (Yes/No)	399(98.8)	6(1.2)
What are the symptoms of TB in human? (fever /swollen lymph glands /respiratory distress/coughing: ≥2 weeks)/ Others) (at least two)	265(65.6)	139(34.4)
How human get infection of TB? (at least two) (consumption of raw/ improper boiled milk/ Contact with infected animal (through aerosol)/Contact with another infected person/butchering of infected animal/ contact/ Consuming raw meat/improper cooked meat of infected animal/ others)	171 (42.3)	233(57.7)
Are animals including wild species, at risk for TB infection? (Yes/No)	146(36.1)	258(63.9)
What are the symptoms of TB in animals? (at least two) (fluctuation of temperature/progressive emaciation/rough hair coat/chronic coughing /presence of dyspnea/ presence of nasal discharge/enlargement of lymph node)	11(2.7)	393(97.3)
Why do you think that your cattle are not at risk of becoming sick by this diseases? (maintain bio-security/ regularly vaccination/ do not let them out of shed/conduct tuberculin skin tests(TST) and to exclude positive animal from the herd)	160(39.6)	244(60.4)
Do you know animal movement/ purchase of a new animal is responsible for TB transmission in your farm? (Yes/No	4(1)	400 (99)
Will the boiling of milk kill the milk borne pathogen including TB bacteria? (Yes/No	161(39.9)	243(60.1)
Is zoonotic TB in human is curable? (Yes/ No)	404(100)	0(0)
What type of treatment is available for TB? (modern/traditional /religious believes)	341(84.4)	63(15.6)
Do you know TB treatment is free of cost available at the DOTS center throughout the country? (Yes/No)	216(53.5)	188(46.5)

**Fig 3:**
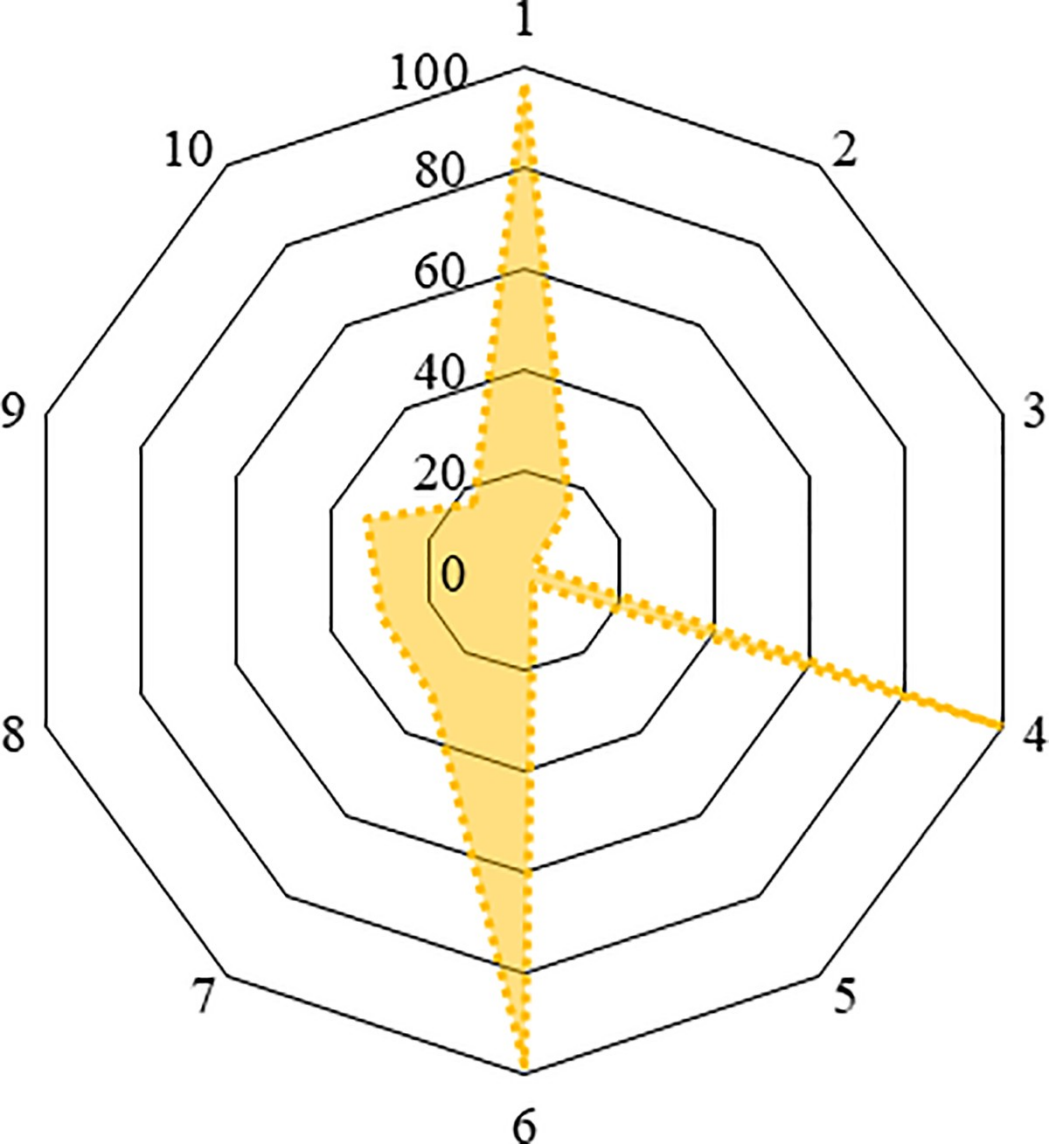
Distribution of appropriate practices among cattle handlers for various questions of the study questionnaire. [(1) What type of milk do you prefer to drink? (raw or unboiled/boiled/both), (2) What do you do when your cattle have been suspected for bTB/zTB? (consult with vets/paravets and do accordingly, sold in market for human consumption), (3) Do you practice separation of TB suspected animals from the healthy animals on your farm? (Yes/No), (4) What type of treatment do you receive if you are infected with zoonotic TB? (modern/ traditional/religious believes), (5) Do you use any protective equipment (mask, gloves, gumboot and apron) while working on the farm? (Yes/No), (6) Do you practice daily cleaning and sanitation practices (regular cleaning cow dung, washing floor, manger, drinker with detergents) on your farm? (Yes/No), (7) Do you smoke or drink or eat food during the handing animals on the farm? (Yes/No), (8) Do you wash your hands and take a shower immediate after working in dairy farm? (Yes/No), (9) Who provides veterinary health care facilities? (Unskilled veterinary practitioner/farmer himself/Para vets/Vets) and (10) Do you share the same premises (where you are living) with animals? (Yes/ No)].

In the final multivariable model, cattle handlers educated at secondary, and higher levels were found to be 2.82- (95% CI: 1.59–5.01) and 5.15- times (95% CI: 1.74–15.20) more likely to have adequate knowledge on zTB control and prevention activities, respectively, compared to those with no formal education. Cattle handlers who reared animals for 1–5 years were more likely (odds ratio (OR) = 2.67, 95% CI: 1.44–4.91) to have adequate knowledge on zTB compared to those who reared animals for >15 years. In addition, cattle handlers with a monthly incomes of 10,000–20,000 taka were significantly (OR = 0.36, 95% CI: 0.14–0.92) less likely to have adequate knowledge on zTB compared to those with a monthly incomes <10,000 taka (Table 4). Nearly one third (31.44%, n = 127) of cattle handlers were found overall to be performing appropriate practices on zTB prevention and control activities (S2 Data). The distribution of appropriate practices among cattle handlers for different questions under survey is presented in Fig 3.

**Table 4:** Predictors of cattle handler’s overall knowledge with respect to zoonotic tuberculosis in the final multivariable logistic regression model.

Variable	Category	Estimate.	SE	Odds ratio (95% Confidence interval)
Education	No formal	Reference	-	1
	Basic/Primary	0.10	0.30	1.10 (0.61–2.01)
	Secondary	1.04	0.29	2.82 (1.59–5.01)
	Higher secondary	0.72	0.43	2.05 (0.87–4.86)
	Graduation and above	1.65	0.55	5.15 (1.74–15.20)
Duration of animal husbandry (years)	1–5	0.98	0.31	2.67 (1.44–4.91)
	6–10	0.51	0.30	1.67 (0.91–3.01)
	11–15	-0.29	0.51	0.74 (0.27–2.01)
	> 15	Reference	-	1
Monthly income (taka)	<10000	Reference	-	1
	10000–20000	-0.99	0.46	0.36 (0.14–0.92)
	20001–50000	-0.03	0.47	0.96(0.38–2.45)
	>50000	0.19	0.51	1.21(0.44–3.36)

Inappropriate practices such as not using protective equipment (97.8%, n = 395); smoking, drinking or eating food (69.1%, n = 279) whilst working with cattle; and sharing the same premises with animals (83.2%, n = 336) were identified (Table 5). Approximately 98.0% (n = 395) of cattle handlers did not separate a zTB suspected animal from others in the herd. Only 3.0% (n = 12) of cattle handlers were found to use personal protective equipment (mask, gloves, gumboot and apron) whilst working in a dairy farm. However, all respondents specified that they would get treatment from a registered physician or government hospital if they were infected with TB. An estimated 67% (n = 272) of the cattle handlers confirmed that veterinary healthcare (treatment and vaccination) provided by unskilled veterinary practitioners or farmers was inappropriate (Table 5). Cattle farmers (29.3%, n = 60) and farm workers (28.1%, n = 18) had a similar level of appropriate practices (Table 6).

**Table 5:** Summary responses on cattle handlers’ practices with respect to zoonotic tuberculosis in the selected districts of Bangladesh (N = 404).

Questions/statements	Practices	
	Appropriate (%)	Inappropriate (%)
What type of milk do you prefer to drink? (raw or unboiled/boiled/both)	394 (97.5)	10 (2.5)
What do you do when your cattle have been suspected for bTB/zTB? (consult vets/paravets and do accordingly, sell in market for human consumption)	64(15.8)	340(84.2)
Do you practice separation of zTB suspected animals from the healthy animals on your farm? (Yes/No)	9(2.2)	395(97.8)
What type of treatment do you receive if you are infected with zTB? (modern/traditional/religious believes)	404(100)	0(0)
Do you use any protective equipment (mask, gloves, gumboot and apron) while working on the farm? (Yes/No)	12(3)	392(97)
Do you practice daily cleaning and sanitation practices (regular cleaning cow dung in stable, washing floor with detergents) in your farm? (Yes/No)	397(98.3)	7(1.7)
Do you smoke or drink or eat food during the handing the animals on the farm? (Yes/No)	125(30.9)	279(69.1)
Do you wash your hands and take a shower immediate after working in dairy farm? (Yes/No)	120 (29.7)	284(70.3)
Who provides veterinary health care facilities? (Unskilled veterinary practitioner/farmer himself/Para vets/Vets)	132(32.7)	272 (67.3)
Do you sharing same premises (where you are living) with animals (yes/ no)	68 (16.8)	336(83.2)

**Table 6:** Association between zoonotic tuberculosis practices with cattle handlers’ demographic and socioeconomic characteristics in the selected districts of Bangladesh.

Variable	Category	Practices		Chi-square P-value
		Inappropriate	Appropriate	
Sex	Male	259(67.6)	124(32.4)	0.13
	Female	18(85.7)	3(14.3)	
Age	20–29	38(61.3)	24(38.7)	0.54
	30–39	72 (66.1)	37(33.9)	
	40–49	74(72.5)	28(27.5)	
	50–59	63(71.6)	25(28.4)	
	>60	30(69.8)	13(30.2)	
Education	No formal	92(75.4)	30(24.6)	<0.001
	Basic/Primary	78(72.2)	30(27.8)	
	Secondary	84 (69.4)	37 (30.6)	
	Higher secondary	17(54.8)	14(45.2)	
	Graduation and above	6 (27.3)	16(72.7)	
Duration of animal rearing (years)	1–5	84(61.8)	52(38.2)	0.002
	6–10	89(65.0)	48(35.0)	
	11–15	20(64.5)	11(35.5)	
	> 15	84(84.0)	16 (16.0)	
Occupation	Others(business man/ shop owner/service holder)	85 (63.4)	49 (36.6)	0.30
	Cattle farmers	146 (70.9)	60 (29.1)	
	Farm worker	46(71.9)	18(28.1)	
Monthly income (BDT)	<10000	23 (82.1)	5 (17.9)	0.09
	10,000–20000	80 (62.0)	49 (38.0)	
	20001–50000	85 (70.8)	35 (29.2)	
	>50000	43 (75.4)	14 (24.6)	

In the final multivariable model of practices, graduation and a higher level of education was found to be significantly (OR = 6.98, 95% CI: 2.47–19.71) associated with appropriate zTB control and prevention practices compared to no formal education. Cattle handlers who reared animals for 1–5 years, 6–10 years and 11–15 years were found to be 2.72- (1.42–5.24), 2.49- (1.29–4.77) and 2.86- times (1.13–7.23) more likely to perform appropriate zTB control and prevention practices compared to those who reared animals for >15 years (Table 7).

**Table 7:** Predictors of cattle handler’s overall practice with respect to zoonotic tuberculosis in the final multivariable logistic regression model.

Variable	Category	Estimate	SE	Odds ratio (95% Confidence Interval)
Education	No formal	Reference	-	1
	Basic/Primary	0.19	0.30	1.21 (0.67–2.21)
	Secondary	0.30	0.29	1.35 (0.75–2.41)
	Higher secondary	0.79	0.42	2.22 (0.97–5.10)
	Graduation and above	1.94	0.52	6.98 (2.47–19.71)
Duration of animal rearing (years)	1–5	1.00	0.33	2.72 (1.42–5.24)
	6–10	0.91	0.33	2.49 (1.29–4.77)
	11–15	1.05	0.47	2.86 (1.13–7.23)
	> 15	Reference	-	1

The Hosmer and Lemeshow goodness of fit test (P-value of 0.28 and 0.69, respectively, for knowledge and practices models) indicated adequate model fit. The accuracy of the final multivariable logistic regression knowledge and practices models were 69.1% (95% CI: 64.3–73.5) and 71.0% (95% CI: 66.4–75.4), respectively, based on k-fold cross-validation result. The area under the ROC curves were 74.1% and 66.6%, respectively, for knowledge and practices models which indicated acceptable predictability to discriminate cattle handlers’ adequate versus inadequate knowledge and appropriate versus inappropriate practices on zTB. No correlation between residuals and predictors and fitted values was observed in either model. Similarly, no outlier was detected (non-significant Bonferonni P-value).

## Discussion

We assessed the knowledge and practices of cattle handlers regarding zTB in some of the promising cattle rearing districts of Bangladesh. Education, duration of animal rearing and monthly income of the cattle handlers were found to be significantly associated with knowledge, whereas education and duration of animal rearing were associated with zTB practices.

Our study findings confirmed that less than half of the respondents (42.6%) have adequate knowledge on zTB control and prevention activities. This finding is in accordance with some earlier studies [41–43]. However, we observed that all categories of cattle handlers were found to have some knowledge on TB in humans relating to its burden, symptoms, treatment options and access to this treatment (Table 2). This finding is consistent with similar studies [44–46]. As a part of the national tuberculosis control program (NTP), TB patients receive treatment from the DOTS centers located throughout the country [14]. Bangladesh is a TB endemic country (3.6% of the total global cases in 2019)[1], which might create awareness about this disease among the stakeholders [47]. This study confirmed that cattle handlers had inadequate knowledge on the mode of transmission, symptoms of zTB in source animals and control measures. Similar findings have also been reported by other researchers [48, 49]. The importance of zTB as part of the national TB burden has been completely ignored in the NTP due to a lack of evidence of the bovine source and because most people consumed boiled milk [14]. However, a study found 6.67% (6/90) of sputum samples to be associated with zTB [50] and another study found 11.11% (2/18) of cases to be zTB positive from sputum, pleural and peritoneal fluid samples collected from humans [16]. Also, *Mycobacterium orygis* as *Mycobacterium tuberculosis* subspecies in source animals such as cattle and captive wild species has been confirmed [4, 51]. Therefore, it is necessary to explore the zTB situation in terms of the national health burden and to develop and disseminate behavioral change communication materials among the targeted risk groups [52, 53]. The majority of respondents (57.7%) had knowledge gaps on the zoonotic transmission of TB in humans. Lack of knowledge about zTB implies that resources need to be found to create public awareness in Bangladesh [54].

The study confirmed several inappropriate practices such as sharing the same premises with animals (83.2%), not washing hands or taking a shower immediately after contact with animals (70.3%), and not wearing protective clothes (97%) which could facilitate the transmission of zTB among cattle handlers [49]. Furthermore, 2.5% of cattle handlers were found to drink raw milk, a risk for the transmission of zTB in humans [55–57]. Moreover, inadequate knowledge about zoonoses was linked with poor animal husbandry practices likely to put cattle handlers at further risk of infection [58].

Our study showed that cattle handlers with a secondary or higher level of education were about three and five times, respectively, more likely to have adequate knowledge. These findings have been confirmed in other geographical locations [59–62]. Furthermore, higher education was found to be linked with appropriate zTB control and prevention practices. This finding suggests that education significantly promotes knowledge as well as practices regarding zTB among animal handlers.

Our study found that those rearing animals for a short period of time were more likely to have adequate knowledge and practices on zTB than those rearing for a long period of time. As a result of different initiatives by the government, educated unemployed youth have begun cattle rearing during the last decade. Therefore, educated cattle handlers with a short (1–5 years) duration of animal rearing were found to be accustomed with appropriate zTB knowledge and practice on control and prevention measures in urban and periurban settings. At present, Bangladesh has become self-sufficient in meat production and aspires to export meat and meat products to other countries in the near future [63].

This study showed that cattle handlers with less monthly income were more likely to have knowledge on zoonotic TB control and prevention activities than those with higher monthly income. Farm workers are mostly paid low (BDT <10,000) salaries but were found to be more knowledgeable than others as they have adopted this profession as their livelihoods. However, higher monthly income (BDT >50,000) is likely to be associated with knowledge on zTB prevention and control activities. This finding is supported by a study conducted in India were a higher household income was consistently linked with reduced risk of TB introduction, as these households were more aware [64].

Zoonotic TB impacts both human and animal sectors, and marginalized communities are the most vulnerable to these impacts [65]. The crossbred cattle population in Bangladesh is gradually increasing in urban and peri-urban areas and most of the cattle rearers are poor and small-scale farmers, and they do not have a formal education. Awareness creation and motivation of target groups with education packages including: zTB transmission, good animal husbandry practices, and personal hygiene are needed to increase animal productivity and reduce public health hazards. The zoonotic impact of this disease is high due to the high human and livestock density at the animal–human interface in both rural and urban settings in the LMICs. In addition, people depend on livestock for their livelihoods [66].

The main limitation of this study was that the cattle handlers were not tested for the presence of zTB. Moreover, slaughter house workers and veterinary practitioners were not included in this study.

## Conclusions

Cattle handlers were found to be have good knowledge on TB in humans, however, they had significant knowledge gaps with respect to zTB in source animals, its transmission dynamics and its risk. Moreover, risky practices might be responsible for the continued zoonotic transmission of tuberculosis in Bangladesh. Creating awareness and motivational training for cattle handlers in these areas under a One Health platform is required.

## Supporting information

S1 Text Field survey questionnaire for the assessment of cattle handlers’ zoonotic TB knowledge and practices in three districts of Bangladesh. (DOCX)

S1 Data Data on cattle handlers’ knowledge with respect to zoonotic TB in three districts of Bangladesh. (XLSX)

S2 Data Data on cattle handlers’ practices with respect to zoonotic TB in three districts of Bangladesh. (XLSX)

S1 STROBE Statement (DOCX)
