# Supplementary material for: Zoonotic tuberculosis knowledge and practices among cattle handlers in selected districts of Bangladesh
Source: PLoS Negl Trop Dis. 2021 Apr 30;15(4):e0009394. doi: 10.1371/journal.pntd.0009394 (PMC8115789; doi:10.1371/journal.pntd.0009394)
Supplement: S1 Text — (DOCX) [file pntd.0009394.s001.docx]

**S1 Text.** Field survey questionnaire for the assessment of cattle handers’ zoonotic TB knowledge and practices in three districts of Bangladesh

**Questionnaire ID:………**

1. **Sociodemographic information**

|  | Geographical location:  Northing: Easting: |  |
| --- | --- | --- |
| Q1 | Location of the farm: Village……..Upazila:……………District: |  |
| Q 2 | Occupation:   - Agriculture (with cattle rearing) - Animal farming/ rearing/ fattening - Animal attendants at the dairy farm - Small shop owner with a cattle farm - Businessman with a cattle farm - Service holder with a cattle farm |  |
| Q3 | Sex of the respondent:   - Male - Female |  |
| Q4 | Age: ………………Years |  |
| Q5 | Religion   - Muslim - Hindu - Buddhist - Christian - Others (specify) |  |
| Q6 | Monthly income (in BDT)  ……………………….. |  |
| Q7 | What is your height education   - No formal education - Basic education(primary (one to five) - Secondary (SSC/equivalent)) - Higher secondary (HSC/equivalent) - Graduation and above |  |
| Q8 | Family size:  …………………… |  |
| Q9 | Duration of cattle rearing:  ……………………Years |  |

1. **Knowledge on zoonotic tuberculosis**

| Q1 | Do you know tuberculosis (TB) can be transmitted from animals to humans or humans to animals as zoonosis?   - Yes - No |  |
| --- | --- | --- |
| Q2 | What is the causal agent of TB?   - Virus - Bacteria - Fungus - Other |  |
| Q3 | Do you know tuberculosis is an important public health problem in Bangladesh?   - Yes - No |  |
| Q4 | What are the symptoms of TB in human?   - Fever - Swollen lymph glands - Respiratory distress/coughing (≥2 weeks) - Others (specify) - Do not know | at least two |
| Q5 | How human get infection of TB?   - Consumption of raw/ improper boiled milk - Contact with infected animal ( through aerosol) - Contact with another infected person - Butchering of infected animal/ contact - Consuming raw meat/improper cooked meat of infected animal - Others (specify) | at least two |
| Q6 | Are animals including wild species, at risk for TB infection?   - Yes - No |  |
| Q7 | What are the symptoms of TB in animals?   - Fluctuation of temperature - Progressive emaciation - Rough hair coat - Chronic coughing - Presence of Dyspnea - Presence of nasal discharge - Enlargement of lymph node | at least two |
| Q8 | Why do you think that your cattle are not at risk of becoming sick by this diseases?   - Maintain bio-security - Regularly vaccinate them - Do not let them out of shed - Conduct tuberculin skin tests(TST) and to exclude positive animal from the herd | at least two |
| Q 9 | Do you know animal movement/ purchase of a new animal is responsible for TB transmission in your farm?   - Yes - No |  |
| Q 10 | Will the boiling of milk kill the milk borne pathogen including TB bacteria?   - Yes - No |  |
| Q 11 | Is zoonotic TB in human is remediable?   - Yes - No |  |
| Q 12 | What type of treatment is available for TB?   - Modern - Traditional - Religious believes |  |
| Q13 | Do you know TB treatment is free of cost available at the DOTS center throughout the country?  -Yes  -No |  |

1. **Practices on zoonotic tuberculosis**

| Q1 | What type of milk do you prefer to drink?   - Raw (unboiled)……….. - Boiled……………………… - Both………………………… |  |
| --- | --- | --- |
| Q2 | What do you do when your cattle have been suspected for bTB/zTB?   - Consult vets/paravets and do accordingly - Sell in market for human consumption |  |
| Q3 | Do you practice separation of zTB suspected animals from the healthy animals on your farm?   - Yes - No |  |
| Q4 | What type of treatment do you receive if you are infected with zTB?   - Modern - Traditional - Religious believes |  |
| Q5 | Do you use any protective equipment (mask, gloves, gumboot and apron) while working on the farm?   - Yes - No |  |
| Q6 | Do you practice daily cleaning and sanitation practices (regular cleaning cow dung in stable, washing floor with detergents) in your farm?   - Yes - No |  |
| Q7 | Do you smoke or drink or eat food during the handing the animals on the farm?   - Yes - No |  |
| Q8 | Do you wash your hands and take a shower immediate after working in dairy farm?   - Yes - No |  |
| Q9 | Who provides veterinary health care facilities?   - Quacks - Farmer himself/herself - Para vets - Vets |  |
| Q10 | Do you sharing same premises (where you are living) with animals?   - Yes - No |  |
