# Supplementary material for: Zoonotic tuberculosis knowledge and practices among cattle handlers in selected districts of Bangladesh
Source: PLoS Negl Trop Dis. 2021 Apr 30;15(4):e0009394. doi: 10.1371/journal.pntd.0009394 (PMC8115789; doi:10.1371/journal.pntd.0009394)
Supplement: S1 STROBE Statement — (DOCX) [file pntd.0009394.s004.docx]

**S1 STROBE Statement**

Checklist of items that should be included in reports of ***cross-sectional studies***

|  | Item No | Recommendation |
| --- | --- | --- |
| **Title and abstract** | 1 | (*a*) Indicate the study’s design with a commonly used term in the title or the abstract **(Line number 20, Page 2)** |
|  |  | (*b*) Provide in the abstract an informative and balanced summary of what was done and what was found **(Section: Abstract, Line number: 23-38, Page 2)** |
| Introduction | | |
| Background/rationale | 2 | Explain the scientific background and rationale for the investigation being reported **Introduction, Line number: 70-97, Page: 4 & 5)** |
| Objectives | 3 | State specific objectives, including any pre-specified hypotheses **(Section: Introduction, Line number: 97-99, Page 5** |
| Methods | | |
| Study design | 4 | Present key elements of study design early in the paper |
| Setting | 5 | Describe the setting, locations, and relevant dates, including periods of recruitment, exposure, follow-up, and data collection **(Section: Materials and methods, Line number: 116-133, Page: 6 & 7)** |
| Participants | 6 | (*a*) Give the eligibility criteria, and the sources and methods of selection of participants **(Section: Materials and methods, Line number: 111-115, Page 6 ) Line number 108-111, Page 5 & 6)** |
| Variables | 7 | Clearly define all outcomes, exposures, predictors, potential confounders, and effect modifiers. Give diagnostic criteria, if applicable **(Section: Materials and methods, Line number: 157-160, Page 8;Line number 168-178, Page 7, 8 & Line number 181-197, Page 9** |
| Data sources/ measurement | 8* | For each variable of interest, give sources of data and details of methods of assessment (measurement). Describe comparability of assessment methods if there is more than one group **(Section: Materials and methods, Line number: 162-197 Page: 8-9)** |
| Bias | 9 | Describe any efforts to address potential sources of bias (N/A) |
| Study size | 10 | Explain how the study size was arrived at **(Section: Materials and methods, Line number: 135-141, Page 7)** |
| Quantitative variables | 11 | Explain how quantitative variables were handled in the analyses. If applicable, describe which groupings were chosen and why **(Line 167-178, Page: 7, 8)** |
| Statistical methods | 12 | (*a*) Describe all statistical methods, including those used to control for confounding  **(Section: Materials and methods, Line number: 167-197, Page 8 & 9** |
|  |  | (*b*) Describe any methods used to examine subgroups and interactions (N/A) |
|  |  | (*c*) Explain how missing data were addressed: N/A |
|  |  | (*d*) If applicable, describe analytical methods taking account of sampling strategy  **(Section: Materials and methods, Line number: 125-129, Page 6** |
|  |  | (*e*) Describe any sensitivity analyses: N/A |
| Results | | |
| Participants | 13* | (a) Report numbers of individuals at each stage of study—eg numbers potentially eligible, examined for eligibility, confirmed eligible, included in the study, completing follow-up, and analysed |
|  |  | (b) Give reasons for non-participation at each stage : N/A |
|  |  | (c) Consider use of a flow diagram |
| Descriptive data | 14* | (a) Give characteristics of study participants (eg demographic, clinical, social) and information on exposures and potential confounders **(Section: Results, Line number: 200-204 Table 1, Page 10-11** |
|  |  | (b) Indicate number of participants with missing data for each variable of interest |
| Outcome data | 15* | Report numbers of outcome events or summary measures **(Section: entire result section Line Number 200-325, Page 10-20** |
| Main results | 16 | (*a*) Give unadjusted estimates and, if applicable, confounder-adjusted estimates and their precision (eg, 95% confidence interval). Make clear which confounders were adjusted for and why they were included **(Section: Results, Line number: 308-313, Page-19; 317-325, Page-20 & Table: 4, 7)** |
|  |  | (*b*) Report category boundaries when continuous variables were categorized **(Section: Materials and Methods, Line number:167-178, Page 8 & 9)** |
|  |  | (*c*) If relevant, consider translating estimates of relative risk into absolute risk for a meaningful time period |
| Other analyses | 17 | Report other analyses done—eg analyses of subgroups and interactions, and sensitivity analyses **(Section: Materials and Methods, Line number:195-197, Page 9)** |
| Discussion | | |
| Key results | 18 | Summarise key results with reference to study objectives **(Section: Discussion, Line number: 360-389, Page 22 & 23)** |
| Limitations | 19 | Discuss limitations of the study, taking into account sources of potential bias or imprecision. Discuss both direction and magnitude of any potential bias **(Section: Discussion, Line number: 390-392, Page 23)** |
| Interpretation | 20 | Give a cautious overall interpretation of results considering objectives, limitations, multiplicity of analyses, results from similar studies, and other relevant evidence **(Section: Discussion, Line number: 331-359, Page: 21, 22 & 381-389, Page:23)** |
| Generalisability | 21 | Discuss the generalisability (external validity) of the study results **(Section: Discussion, Line number: 381-389, Page 23)** |
| Other information | | |
| Funding | 22 | Give the source of funding and the role of the funders for the present study and, if applicable, for the original study on which the present article is based  **N/A (included in the submission system)** |

*Give information separately for exposed and unexposed groups.

**Note:** An Explanation and Elaboration article discusses each checklist item and gives methodological background and published examples of transparent reporting. The STROBE checklist is best used in conjunction with this article (freely available on the Web sites of PLoS Medicine at http://www.plosmedicine.org/, Annals of Internal Medicine at http://www.annals.org/, and Epidemiology at http://www.epidem.com/). Information on the STROBE Initiative is available at www.strobe-statement.org.
